# Books of Interest to Nurses

**Published:** 1920-10-09

**Authors:** 


### BOOKS OF INTEREST TO NURSES.

**HEALTH READER FOR GIRLS.** By Agnes L. Stenhouse and C. Stenhouse, B.Sc. (London: Macmillan and Co., Ltd. Price 3s. net.)

The authors of this book* seem to have attempted the impossible, and to have come within measurable distance of success. That is to say, they have tried to deal with the whole question of healthy living in a manual five inches by seven, of 186 pages, with good type and numerous illustrations. The index is in itself surprising, we take at random Boots, Boracic Acid, Brain, Bread, Breathing, Economy (of Food), Eggs, Enamel of Tooth, Energy, Exercise. Comparison with the early health reading-books shows the immense advance made towards a scheme of teaching really applicable to the needs of the ordinary young person. The earliest books, and many much later ones, seemed to have in view the training of incipient doctors rather than that of the ordinary citizen, and began with such detailed anatomy teaching that the schoolmistresses were troubled by many remonstrances from mothers to the effect that these lessons were "rude."

The present book begins also with a little anatomy, both in text and illustration, but it is entirely of a practical kind, calculated to show to young people the value of good physical habits and the danger of bad ones. The emphasis laid upon unwholesome postures in a picture of a girl lounging while reading and one of a mother pushing a child in a chair where it sleeps hunched up should teach useful lessons to mothers as well as to girls. The present writer confesses to a doubt as to whether it is wise to teach young people about calories, protein, carbohydrates and the like, especially with the aid of diagrams. But a book of this kind is likely to be used rather as a text-book for teachers than as a reader throughout the school. Its price, three shillings, would make the question of cost difficult in many cases. The chapter on Pure Air, therefore, or that on cleanliness, which delightfully shows a fly as "the most dangerous wild animal in England," might very well be used by the teacher as a foundation for a lesson, and the illustrations would in many cases serve excellently as blackboard illustrations. We might especially suggest those on pages 120 and 121, which show the formation of the feet and illustrate the criminal form of many fashionable shoes. Critics will no doubt take exception to some statements. The authors, for instance, maintain the heresy of a midnight feed for infants, and mothers may be a little dismayed if their family demands a spoonful of jam or marmalade in its glasses of drinking-water. But generally the advice given is practical and suited to the conditions of everyday life, and the book is, in spite of its concentration, very pleasant to read.

**Medical Gymnastics in Medicine and Surgery.** By E. Bellis Clayton, M.B., B.C. (Cantab) (London: Edward Arnold. Price 5s. net.)

The author's object in writing this text-book was to place in the student's hands a guide to the rational application of exercises and manipulations to the various diseases and morbid conditions which yield to or are benefited by physio-therapeutic treatment. The trained masseuse, even of to-day, does not invariably display that thoughtful discretion in her work upon which so much depends. Skill in the technique of manipulations is futile if the aims and effects of the treatment are not properly appreciated. The successful masseuse is the one who has realised the need for acquiring a common-sense knowledge of the condition she treats. It is possible that dependence upon school notes for this side of training has handicapped many students, but whether this be so or otherwise, we are confident that this book will be welcomed by students and practitioners alike. The subject-matter is arranged methodically, the various systems referred to being grouped under their respective systems. The opening chapter on general pathology and the principles of treatment, though somewhat condensed, contain much useful information set out in a clear and practical manner. The chapters on fractures and injuries and diseases of the joints are exceptionally valuable, and the appendices contain some carefully-drawn schemes of exercises and instruction in the general principles of the re-education of active movements. The labour involved in the production of the text-book is amply justified, and the work might be adopted with advantage by training-schools and teachers.

**A Scottish Nurse at Work.** Being a record of what one semi-trained nurse has been privileged to see and do during four and a-half years of war. With seven illustrations. By Henrietta Tayler. (John Lane: The Bodley Head. Price 5s. net.)

Few more modest and interesting records of war experiences have come into our hands than these concisely put together reminiscences of the Scottish nurse. They include service in Scotland under the Red Cross, at La Panne under the famous Dr. Depage, at Adinkerke with refugee Belgian children, in a hospital for rapatriés on the borders of Switzerland, in Florence at a military hospital, on the Italian Front in a French ambulance and in tents among Austrian prisoners, again in tents among prisoners of all nationalities, in another French ambulance, and finally in Gorizia. Everywhere Miss Tayler had an eye for the humorous, an ear for linguistic variety, and a large heart for patients of strangely mixed nationality. The names conferred on her by her patients are evidence of the races she tended: Liebe Schwester, Gnädigs Fräulein, Schönerfrau, Gospodja, Sestra, Maika, Mámá, Mummy, Màdama, and finally Mutter. The comments on hospital administration among our Allies are always good-humoured, but we are told that the duties of the Red Cross nurses not infrequently consist in washing the faces of those who have been unwashed for weeks. "Even in a French hospital one sick man has been heard to say to a newly-arrived friend, 'On te lave les pieds ici, mon vieux, et cela fait tellement de bien.'"

**Rhoda Drake.** By C. H. Dudley Ward, D.S.O., M.C. (John Murray. Price 7s. net.)

This is a clever story, well conceived and executed, of the new sort of new woman, amazingly well able to take care of herself, full of strange insight into other people's minds, but a primitive woman all the time, with the wholesome conviction that life begins and ends with a baby. Rhoda Drake, we could not help reflecting, would have made a capital nursing sister. The manner in which she gets the better of a spy woman, handles a weak husband, and helps to unveil a very subtle plot proves her of the stuff which makes heroines both in fiction and real life.

